# First-in-human, open-label, phase 1/2 study of the monoclonal antibody programmed cell death protein-1 (PD-1) inhibitor cetrelimab (JNJ-63723283) in patients with advanced cancers

**DOI:** 10.1007/s00280-022-04414-6

**Published:** 2022-03-17

**Authors:** Enriqueta Felip, Victor Moreno, Daniel Morgensztern, Giuseppe Curigliano, Piotr Rutkowski, José Manuel Trigo, Aitana Calvo, Dariusz Kowalski, Diego Cortinovis, Ruth Plummer, Michele Maio, Paolo A. Ascierto, Vladimir I. Vladimirov, Andres Cervantes, Enrique Zudaire, Anasuya Hazra, Huybrecht T’jollyn, Nibedita Bandyopadhyay, James G. Greger, Edward Attiyeh, Hong Xie, Emiliano Calvo

**Affiliations:** 1 Thoracic Cancer Unit, Oncology Department, Vall d’Hebron University Hospital, Vall d’Hebron Institute of Oncology, Barcelona, Spain; 2 Phase 1 Trials Unit, START MADRID-FJD, Hospital Fundación Jiménez Díaz Medical Oncology Division, Madrid, Spain; 3 Division of Oncology, Section of Medical Oncology, Washington University School of Medicine, St. Louis, MO USA; 4 Division of Early Drug Development, European Institute of Oncology, IRCCS and University of Milano, Milan, Italy; 5 Department of Soft Tissue/Bone Sarcoma and Melanoma, Maria Skłodowska-Curie National Research Institute of Oncology, Warsaw, Poland; 6 Department of Medical Oncology, Hospital Universitario Virgen de La Victoria y Regional, Malaga, Spain; 7 Oncology Service, Hospital General Universitario Gregorio Maranon, Madrid, Spain; 8 Oncology Unit, San Gerardo Hospital, Monza, Italy; 9 Sir Bobby Robson Unit, Northern Centre for Cancer Care, Newcastle Hospitals NHS Trust and Newcastle University, Newcastle, UK; 10 Center for Immuno-Oncology, Azienda Ospedaliera Universitaria Senese, University of Siena, Siena, Italy; 11 Unit of Melanoma, Cancer Immunotherapy and Development Therapeutics, Istituto Nazionale Tumori IRCCS–Fondazione Pascale, Napoli, Italy; 12 Pyatigorsky Oncology Dispensary, Pyatigorsk, Russia; 13 Medical Oncology Department, INCLIVA Biomedical Research Institute, University of Valencia, Valencia, Spain; 14 Janssen Research & Development, Spring House, PA USA; 15 Janssen Research & Development, Beerse, Belgium; 16 Janssen Research & Development, Raritan, NJ USA; 17 Centro Integral Oncológico Clara Campal Medical Oncology Division, START Madrid-CIOCC, Sanchinarro University Hospital, Madrid, Spain

**Keywords:** Monoclonal antibody PD-1 inhibitor efficacy, Non-small-cell lung cancer, Melanoma, Colorectal cancer, Microsatellite instability–high, Pharmacokinetics/pharmacodynamics

## Abstract

**Purpose:**

To assess the safety, pharmacokinetics, pharmacodynamics, and preliminary efficacy of cetrelimab (JNJ-63723283), a monoclonal antibody programmed cell death protein-1 (PD-1) inhibitor, in patients with advanced/refractory solid tumors in the phase 1/2 LUC1001 study.

**Methods:**

In phase 1, patients with advanced solid tumors received intravenous cetrelimab 80, 240, 460, or 800 mg every 2 weeks (Q2W) or 480 mg Q4W. In phase 2, patients with melanoma, non-small-cell lung cancer (NSCLC), and microsatellite instability–high (MSI-H)/DNA mismatch repair-deficient colorectal cancer (CRC) received cetrelimab 240 mg Q2W. Response was assessed Q8W until Week 24 and Q12W thereafter.

**Results:**

In phase 1, 58 patients received cetrelimab. Two dose-limiting toxicities were reported and two recommended phase 2 doses (RP2D) were defined (240 mg Q2W or 480 mg Q4W). After a first dose, mean maximum serum concentrations (*C*_max_) ranged from 24.7 to 227.0 µg/mL; median time to *C*_max_ ranged from 2.0 to 3.2 h. Pharmacodynamic effect was maintained throughout the dosing period across doses. In phase 2, 146 patients received cetrelimab 240 mg Q2W. Grade ≥ 3 adverse events (AEs) occurred in 53.9% of patients. Immune-related AEs (any grade) occurred in 35.3% of patients (grade ≥ 3 in 6.9%). Overall response rate was 18.6% across tumor types, 34.3% in NSCLC, 52.6% in programmed death ligand 1–high (≥ 50% by immunohistochemistry) NSCLC, 28.0% in melanoma, and 23.8% in centrally confirmed MSI-H CRC.

**Conclusions:**

The RP2D for cetrelimab was established. Pharmacokinetic/pharmacodynamic characteristics, safety profile, and clinical activity of cetrelimab in immune-sensitive advanced cancers were consistent with known PD-1 inhibitors.

**Trial registrations:**

NCT02908906 at ClinicalTrials.gov, September 21, 2016; EudraCT 2016–002,017-22 at clinicaltrialsregister.eu, Jan 11, 2017.

**Supplementary Information:**

The online version contains supplementary material available at 10.1007/s00280-022-04414-6.

## Introduction

The development of immune checkpoint inhibitors led to improved outcomes and expanded opportunities for targeted combination therapies in numerous tumor types [1]. The programmed cell death protein-1 (PD-1) is an immune checkpoint receptor that regulates adaptive T cell immunity. PD-1 is expressed on activated CD4^+^ and CD8^+^ T cells and suppresses T cell function when bound to its ligands, programmed death ligand 1 (PD-L1) and 2 (PD-L2). In the tumor microenvironment, PD-1 activity can suppress tumor immunosurveillance and development of adaptive immune responses [1, 2]. Hence, blocking PD-1 receptor–ligand interactions can enhance antitumor immune responses to tumor cells.

Monoclonal antibody PD-1 inhibitors such as pembrolizumab [3, 4], nivolumab [5, 6], and cemiplimab [7, 8] have been approved by the US Food and Drug Administration and the European Medicines Agency based on durable responses in immune-sensitive cancers. Other PD-1 inhibitors have been approved for various cancer treatment indications in different geographic regions.

Several biomarkers have been identified to be predictive of response to PD-1 antagonists. Nivolumab and pembrolizumab have demonstrated higher response rates in solid tumors in which > 1% of cells are PD-L1 + by immunohistochemistry (IHC) compared with tumors with < 1% PD-L1 positivity [9–11]. Tumor cells deficient in mismatched DNA repair systems (dMMR) are responsive to these agents and microsatellite instability (MSI) has proven to be a marker for dMMR [12–15].

Cetrelimab is a fully human immunoglobulin G4 monoclonal antibody inhibitor of PD-1. The in vitro binding affinity to the human PD-1 extracellular domain for cetrelimab is *K*_D_ = 1.72 nM [16] compared with pembrolizumab at *K*_D_ = 29 pM [17, 18] and nivolumab at *K*_D_ = 3.06 nM [18, 19]. Like nivolumab and pembrolizumab, cetrelimab completely inhibits binding of PD-1 to PD-L1 and PD-L2. All three PD-1 inhibitors have demonstrated dose-dependent induction of interferon (IFN)-γ, tumor necrosis factor-α, and interleukin (IL)-4 upon cytomegalovirus (CMV) stimulation of CMV-reactive T cells from peripheral blood of CMV-responsive donors or CD4^+^ T cells that were activated by stimulation with allogeneic, major histocompatibility complex–mismatched, dendritic cells [18, 20]. Like nivolumab [20] and pembrolizumab [18], cetrelimab achieved tumor growth inhibition of MC38 tumors implanted on human PD-1 knock-in mice (companion paper by DeAngelis et al. in this issue of *Cancer Chemotherapy and Pharmacology*).

The first-in-human phase 1/2 LUC1001 study was designed to evaluate the safety, pharmacokinetics, pharmacodynamics, and clinical activity of cetrelimab in patients with advanced solid tumors.

## Materials and methods

### Study design

**Fig. 1:**
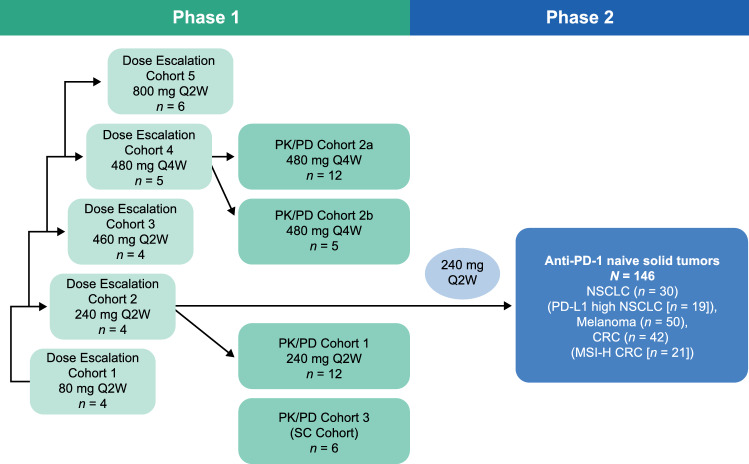
LUC1001 study design. Unless specified, route of administration was IV. PK/PD Cohort 2a included 12 patients who received the lyophilized formulation; all other cohorts received the frozen liquid formulation. *CRC* colorectal cancer, *MSI-H* microsatellite instability–high, *NSCLC* non-small-cell lung cancer, *PK/PD* pharmacokinetics/pharmacodynamics, *Q2W* every 2 weeks, *Q4W* every 4 weeks, *IV* intravenous, *PD-L1* programmed death ligand 1, *SC* subcutaneous

LUC1001 (NCT02908906, EudraCT 2016–002,017-22) was an open-label, multicenter phase 1/2 study of cetrelimab (Fig. 1) initiated in Nov 2016. LUC1001 was conducted in accordance with the International Council for Harmonisation Good Clinical Practice Standards and the Declaration of Helsinki. The protocol was approved by institutional and ethics committees. All patients provided written informed consent.

### Patients

All patients enrolled in LUC1001 were required to be aged ≥ 18 years, with metastatic or unresectable solid tumors, and had progressed on or been deemed ineligible for standard antitumor therapy. Patients were required to have Eastern Cooperative Oncology Group performance status ≤ 1 and to have had no prior treatment with PD-1/PD-L1 inhibitors.

Patients with any solid tumor except lymphoma were eligible to enroll in the phase 1 part of LUC1001. Tissue sample collection for PD-L1 testing was optional in phase 1 but mandatory in phase 2. The phase 2 part of LUC1001 was initially designed to enroll patients with histologically or cytologically confirmed stage III or IV PD-L1^+^ [≥ 1% PD-L1^+^ on tumor cells assessed centrally with the 22C3 antibody (Dako Omnis; Agilent, Santa Clara, CA) or local testing] non-small-cell lung cancer (NSCLC), melanoma, or colorectal cancer (CRC), as well as renal cell carcinoma, bladder cancer, small-cell lung cancer (SCLC), and gastric/esophageal cancer. The protocol was amended to limit phase 2 recruitment to PD-L1-high (≥ 50% PD-L1^+^ on tumor cells) NSCLC, melanoma, and MSI-high (MSI-H)/dMMR CRC determined by local or central testing. MSI-H status was centrally confirmed retrospectively using the Promega fluorescent PCR-based MSI Analysis System v1.2 (Promega Corporation, Madison, WI). For both PD-L1 status in NSCLC and MSI-H status in CRC, only central testing data were used in this analysis.

### Study treatments

Phase 1 included five dose-escalation cohorts and three pharmacokinetics/pharmacodynamics cohorts; patients received intravenous (IV) 80, 240, 460, or 800 mg every 2 weeks (Q2W) or 480 mg Q4W doses of cetrelimab (Fig. 1). Additionally, feasibility of subcutaneous (SC) administration of cetrelimab was, and continues to be, explored. SC injection was administered by slow manual push. All patients in phase 1 and 2 received a frozen liquid formulation except pharmacokinetics/pharmacodynamics Cohort 2a, in which 12 patients received the lyophilized formulation at 480 mg Q4W, and pharmacokinetics/pharmacodynamics Cohort 3 (SC cohort), in which six patients received the lyophilized formulation by SC injection. The first IV infusion was delivered over 60 min. If the 60-min infusion was well tolerated, subsequent infusions could be delivered over 30 min.

Because of limited information due to the small sample size, pharmacokinetic data in the SC cohort are not reported here; these patients were included with the appropriate tumor-specific groups in efficacy assessments and with the IV 480 mg Q4W dose group in safety assessments.

#### Dose escalation

Decisions about dose escalation were based on the rate of dose-limiting toxicities (DLTs) observed during the first 28 days on treatment. DLTs were defined as toxicities of grade 5, grade 4 (including neutropenia lasting ≥ 7 days and thrombocytopenia of any duration), or grade ≥ 3 [with the exception of asthenia, fever, constipation, fatigue that improves in ≤ 7 days, nausea lasting for ≤ 7 days with standard of care, vomiting or diarrhea lasting ≤ 3 days with standard care, tumor flare (local pain, irritation, or rash at known or suspected tumor sites) that improves in ≤ 7 days, aspartate aminotransferase/alanine aminotransferase (AST/ALT) elevation lasting < 7 days, laboratory abnormalities not requiring hospitalization and deemed not clinically significant by the investigator, and thrombocytopenia without clinically significant bleeding]. ALT/AST elevations meeting Hy’s law criteria and immune-related toxicities requiring treatment in excess of corticosteroids were included in the DLT definition.

#### Cohort expansion

The clinical activity and safety of cetrelimab at the chosen RP2D in three tumor-specific groups (NSCLC, melanoma, and MSI-H/dMMR CRC) were assessed in phase 2 to confirm the selected doses.

### Study endpoints

The primary endpoints were the safety and tolerability, and overall response rate (ORR) of cetrelimab. Secondary objectives included assessment of pharmacokinetics, pharmacodynamics, immunogenicity, and efficacy by the investigator, including the clinical benefit rate (CBR), progression-free survival (PFS), overall survival (OS), and duration of response (DOR). Selected biomarkers were examined for potential association with pharmacodynamic modulation of cetrelimab.

### Safety assessments

Safety assessments included the frequency and severity of treatment-emergent adverse events (TEAEs) and immune-related adverse events (irAEs), infusion-related reactions (IRRs), vital sign measurements, clinical laboratory values, and electrocardiograms. The severity of AEs was assessed using the National Cancer Institute Common Terminology Criteria for Adverse Events v4. irAEs and IRRs were designated by the investigators. Investigators were instructed to consider all events of an inflammatory nature immune related in the absence of a clear alternative etiology.

### Pharmacokinetics and receptor occupancy

Blood samples were collected for pharmacokinetic analyses on the day of dosing both pre-infusion (within 2 h) and at the end of infusion (EOI) for the first ten doses in cohorts receiving Q2W dosing. After Dose 1 and Dose 9, additional blood samples were collected at 2 h (EOI + 2 h) and 6 h (EOI + 6 h) post infusion as well as on Days 2, 4, and 8 in the cohorts receiving Q2W dosing. After Dose 1 and Dose 5 in cohorts receiving Q4W dosing, additional samples were collected at EOI + 2 h and EOI + 6 h post infusion as well as on Days 2, 4, 8, 15, and 22. In the SC cohort, blood samples were collected for the sentinel SC dose at the following time points: pre-injection (within 2 h), post injection (PI), PI + 2 h, and PI + 6 h, as well as on Days 2, 4, 8, 15, 22, 29, and 36. Samples were also collected at the end-of-treatment (EOT) visit (≤ 30 days after the last dose) and first survival follow-up visit (~ 12 weeks after the last dose).

Serum cetrelimab concentrations were measured using a validated electrochemiluminescence immunoassay method. As previously mentioned, two drug product formulations were tested at the 480 mg Q4W dose.

Individual pharmacokinetic parameters were calculated using noncompartmental analysis (NCA) and descriptive statistics were provided. A two-compartmental disposition population pharmacokinetic (popPK) model with first-order elimination and zero-order IV infusion rate, parameterized in terms of clearance (CL), volume of central compartment (V1), intercompartmental CL (Q), and volume of peripheral compartment (V2), was used to describe the pharmacokinetics of cetrelimab from both phase 1 and phase 2 parts of LUC1001. Interindividual variability (IIV) was implemented on CL, V1, and V2, with random-effects correlation estimated between CL and V1. IIV was also included on residual error to allow for patient-level variations in residual error. Additionally, popPK modeling and simulations were performed using data from all dose cohorts to explore multiple phase 2 dosing regimens targeting comparable pharmacokinetic profiles and exposures similar to nivolumab and pembrolizumab.

Fresh whole blood was collected from all patients in phase 1 and from the first 40 patients in phase 2 for evaluation of PD-1 receptor occupancy (RO) on circulating CD3^+^ T cells by flow cytometry analysis. During phase 1, Dose 1 samples were taken on Day 1 before infusion and 2 h after EOI and on Day 8; Dose 2 samples were taken on Day 1 before infusion and at EOI. Samples for Dose 3 were taken on Day 1 before infusion, and for Dose 9 on Day 1 before infusion and at EOI. Dose 10 samples were drawn on Day 1 at EOI and at EOT. During phase 2, blood samples were taken before infusion on Dose 1 and Dose 5.

### Pharmacodynamics and biomarkers

Ex vivo staphylococcal enterotoxin B (SEB) stimulation of IL-2 production by peripheral blood mononuclear cells was conducted to evaluate cetrelimab pharmacodynamic modulation [21]. Briefly, whole blood samples were diluted 1:10 with RPMI 1640 medium (Catalog #111,875,093, Thermo Fisher Scientific, Waltham, MA) followed by a 4-day incubation with 100 ng/mL of SEB and 10 µg/mL of cetrelimab or isotype control. The ratio of IL-2 expression levels between the isotype and cetrelimab ex vivo-treated blood samples was then calculated to assess the degree of pharmacodynamic modulation, with a ratio of 1 indicating maximum T cell activation.

Serum levels of IFN-γ-inducible protein 10 (IP10) and IL-2 receptor alpha chain (IL2Ra) were measured by Meso Scale Discovery (MSD) for all patients with one pretreatment and at least one post-treatment sample collection.

### Immunogenicity

Serum samples were screened for anti-drug antibodies binding to cetrelimab and the titer of confirmed positive samples was reported.

### Efficacy assessments

Tumor response was assessed by the investigators per Response Evaluation Criteria In Solid Tumors (RECIST) v1.1 [22] every 8 weeks (± 2 weeks) until Week 24, after which assessments were made every 12 weeks (± 2 weeks). Patients were allowed to continue treatment with study drug beyond initial radiologic tumor progression determined on the basis of the RECIST criteria. This allowance took into account the observation that some patients can have a transient tumor flare (i.e., pseudo-progression) in the first few months after the start of immunotherapy but can develop subsequent disease response. Patients were advised to continue study treatment at the discretion of the treating physician while waiting for confirmation of disease progression if they were clinically stable as defined by the following criteria: (1) absence of clinical signs and symptoms indicating disease progression; (2) clinical disease progression not requiring immediate therapeutic intervention; (3) no decline in Eastern Cooperative Oncology Group performance status; and (4) absence of progressive tumor at critical anatomical sites (e.g., cord compression) requiring urgent alternative medical intervention. Patients who were deemed clinically unstable could discontinue study treatment prior to repeat imaging for confirmation of progressive disease.

ORR was defined as the percentage of patients with complete response (CR) + partial response (PR). CBR was defined as the percentage of patients with CR + PR + stable disease lasting ≥ 24 weeks [23]. PFS was defined as the time from first dose of cetrelimab to progressive disease or death due to any cause. OS was defined as the time from first dose of cetrelimab to death due to any cause. DOR was defined as the time from initial response of CR or PR to progressive disease or death due to underlying disease.

### Statistical analysis

Dose escalation and recommended phase 2 dose identification were guided using a modified continual reassessment method, which was based on the probability of dose-limiting toxicities by a two-parameter Bayesian logistic regression model and escalation with overdose control principle. The sample size estimation of 180 patients for the overall study population in the dose expansion part of this study was based on the ability to detect a 79.0% success rate if the true ORR is 15.0% and 15.7% if the true ORR is 10.0% by Bayesian power, or a 43.2% success rate if the true ORR is 15.0% and 1.3% if the true ORR is 10% by Bayesian double criteria. The all-treated population, defined as patients who received ≥ 1 dose of cetrelimab, was the basis for both safety and efficacy analyses. Subpopulations of patients with NSCLC, melanoma, and CRC were analyzed individually. The ORR is presented with 2-sided 95% exact Clopper–Pearson confidence intervals (CIs). Time-to-event endpoints and corresponding 95% CIs were estimated using Kaplan–Meier methodology.

## Results

### Patients

At clinical data cutoff on Jul 1, 2019, the all-treated population comprised 204 patients (58 in phase 1 and 146 in phase 2). Median age was 60.0 years (range, 23.0–86.0 years) and 56.9% were male (Table 1). The majority of patients (70.1%) had previously received ≥ 2 regimens.

**Table 1:** Demographics and disease characteristics at baseline (all-treated population)

	NSCLC *n* = 35	MEL *n* = 50	BC *n* = 4	RCC *n* = 2	SCLC *n* = 12	MSI-H /dMMR CRC *n* = 48	Gastric/esophageal *n* = 16	Other^a^ *n* = 37	Total *N* = 204
Median age, years (range)	64.0 (47.0–79.0)	60.5 (23.0–86.0)	64.0 (56.0–79.0)	71.0 (66.0–76.0)	63.0 (46.0–80.0)	59.5 (29.0–81.0)	62.0 (44.0–82.0)	53.0 (27.0–80.0)	60.0 (23.0–86.0)
Sex, *n* (%)									
Male	29 (82.9)	29 (58.0)	3 (75.0)	1 (50.0)	8 (66.7)	20 (41.7)	10 (62.5)	16 (43.2)	116 (56.9)
Female	6 (17.1)	21 (42.0)	1 (25.0)	1 (50.0)	4 (33.3)	28 (58.3)	6 (37.5)	21 (56.8)	88 (43.1)
ECOG PS, *n* (%)									
0	13 (37.1)	29 (58.0)	2 (50.0)	0 (0)	3 (25.0)	27 (56.3)	8 (50.0)	20 (54.1)	102 (50.0)
1	22 (62.9)	21 (42.0)	2 (50.0)	2 (100.0)	9 (75.0)	21 (43.8)	8 (50.0)	17 (45.9)	102 (50.0)
Previous cancer therapy									
Surgery/procedure	20 (57.1)	49 (98.0)	4 (100.0)	1 (50.0)	3 (25.0)	48 (100.0)	11 (68.8)	36 (97.3)	172 (84.3)
Radiotherapy	19 (54.3)	20 (40.0)	0	1 (50.0)	11 (91.7)	14 (29.2)	7 (43.8)	22 (59.5)	94 (46.1)
Systemic therapy	35 (100.0)	37 (74.0)	4 (100.0)	2 (100.0)	12 (100.0)	48 (100.0)	16 (100.0)	37 (100.0)	191 (93.6)
Number of prior lines of regimens, *n* (%)									
0	0 (0)	13 (26.0)	0 (0)	0 (0)	0 (0)	0 (0)	0 (0)	0 (0)	13 (6.4)
1	18 (51.4)	12 (24.0)	1 (25.0)	1 (50.0)	6 (50.0)	2 (4.2)	3 (18.8)	5 (13.5)	48 (23.5)
2	12 (34.3)	15 (30.0)	2 (50.0)	0 (0)	4 (33.3)	19 (39.6)	6 (37.5)	8 (21.6)	66 (32.4)
≥ 3	5 (14.3)	10 (20.0)	1 (25.0)	1 (50.0)	2 (16.7)	27 (56.3)	7 (43.8)	24 (64.9)	77 (37.7)

*BC* bladder cancer, *CRC* colorectal cancer, *dMMR* DNA mismatch repair deficient, *ECOG PS* Eastern Cooperative Oncology Group performance status, *MEL* melanoma, *MSI-H* microsatellite instability–high, *NSCLC* non-small-cell lung cancer, *RCC* renal cell carcinoma, *SCLC* small-cell lung cancer

^a^Any other type of advanced or refractory solid tumor malignancy, except lymphoma, that was metastatic or unresectable (e.g., breast cancer, prostate cancer, or pancreatic adenocarcinoma)

### RP2D determination

Cetrelimab 240 mg Q2W and 480 mg Q4W resulted in the same total dose, while demonstrating sufficient pharmacokinetics/pharmacodynamics coverage to ensure RO saturation throughout the dosing interval. Therefore, the RP2D for cetrelimab may be administered as either 240 mg Q2W or 480 mg Q4W; 240 mg Q2W was selected as the RP2D for the phase 2 part of the study.

### Safety

During the phase 1 dose escalation, two DLTs were reported. Both events were considered serious and possibly treatment related. One patient with NSCLC receiving 240 mg Q2W experienced a DLT of grade 3 pleural effusion that resulted in treatment interruption. This patient received seven additional cetrelimab doses after treatment interruption before cetrelimab treatment was discontinued due to disease progression. A second patient with metastatic thymoma receiving 800 mg Q2W experienced a DLT of grade 5 myasthenia gravis, 16 days after receiving Dose 1. Prior treatment for thymoma included four lines of chemotherapy or investigational compounds, radiation for phrenic nerves and pericardial infiltration, and extensive surgical procedures of hemidiaphragm and ipsilateral pericardium excision, left hemithorax pleurectomy, left thoracotomy, myoplasty, pericardial and diaphragmatic replacement, prosthesis placement, tumor resection (thymoma), and posterolateral thoracotomy. The symptom for myasthenia gravis was grade 4 dyspnea and antiacetylcholine receptor antibody was positive. The patient received methylprednisolone 60 mg (1 mg/kg) twice daily and ipratropium bromide, along with noninvasive ventilation; however, diaphragmatic sequelae from multiple chest surgeries limited supportive respiratory therapy options.

The most frequently reported TEAEs (occurring in > 15.0% of patients) were asthenia (25.5%), fatigue (21.1%), dyspnea (21.1%), pyrexia (19.6%), diarrhea (19.1%), anemia (18.6%), nausea (17.6%), decreased appetite (17.6%), cough (17.2%), and back pain (15.7%). Approximately two-thirds of patients (67.2%) experienced TEAEs deemed possibly treatment related (Table 2). Serious treatment-related TEAEs occurred in 10.8% of patients and grade ≥ 3 treatment-related TEAEs occurred in 13.7%.

**Table 2:** Summary of TEAEs (all-treated population)

	80 mg Q2W *n* = 4	240 mg Q2W *n* = 162	460 mg Q2W *n* = 4	480 mg Q4W *n* = 28	800 mg Q2W *n* = 6	Total *N* = 204
Any TEAEs, *n* (%)						
Any	4 (100.0)	158 (97.5)	4 (100.0)	27 (96.4)	6 (100.0)	199 (97.5)
Treatment related	2 (50.0)	115 (71.0)	3 (75.0)	13 (46.4)	4 (66.7)	137 (67.2)
Serious TEAEs, *n* (%)						
Any	3 (75.0)	79 (48.8)	3 (75.0)	12 (42.9)	5 (83.3)	102 (50.0)
Treatment related	0 (0)	20 (12.3)	0 (0)	0 (0)	3 (33.3)	22 (10.8)
Grade ≥ 3 TEAEs, *n* (%)						
Any	2 (50.0)	87 (53.7)	2 (50.0)	14 (50.0)	5 (83.3)	110 (53.9)
Treatment related	0 (0)	24 (14.8)	0 (0)	2 (7.1)	3 (33.3)	28 (13.7)
TEAEs leading to treatment interruption, *n* (%)						
Any	2 (50.0)	85 (52.5)	4 (100.0)	5 (17.9)	4 (66.7)	100 (49.0)
Treatment related	0 (0)	47 (29.0)	1 (25.0)	1 (3.6)	3 (50.0)	52 (25.5)
TEAEs leading to dose reduction, *n* (%)						
Any	0 (0)	0 (0)	0 (0)	0 (0)	0 (0)	0 (0)
Treatment related	0 (0)	0 (0)	0 (0)	0 (0)	0 (0)	0 (0)
TEAEs leading to withdrawal, *n* (%)						
Any	0 (0)	17 (10.5)	0 (0)	1 (3.6)	1 (16.7)	19 (9.3)
Treatment related	0 (0)	11 (6.8)	0 (0)	0 (0)	1 (16.7)	12 (5.9)
TEAEs leading to death, *n* (%)						
Any	0 (0)	13 (8.0)	0 (0)	0 (0)	2 (33.3)	15 (7.4)
Treatment related	0 (0)	0 (0)	0 (0)	0 (0)	1 (16.7)	1 (0.5)

Patients with > 1 record are counted only once at corresponding rows

*Q2W* every 2 weeks, *Q4W* every 4 weeks, *TEAE* treatment-emergent adverse event

Twenty-one patients (10.3%) died within 30 days of their last cetrelimab dose. For 17 patients, the primary cause of death was disease progression. AEs were the primary cause of death for four patients and one of those AEs (the DLT of myasthenia gravis) was considered treatment related. Dyspnea was the cause of death in two patients and acute heart failure in one patient. Fifteen patients (7.4%) experienced TEAEs leading to death.

The safety profile at the RP2D (240 mg Q2W IV) was similar to that in the overall all-treated population, with serious and grade ≥ 3 TEAEs that were considered treatment-related occurring in 12.3% and 14.8%, respectively. Treatment-related TEAEs at a dose of 240 mg Q2W resulted in dose interruptions in 29.0% of patients and discontinuation in 6.8%.

### AEs of clinical interest

Immune-related AEs were reported by the investigator for 35.3% of patients (72/204). The most common irAEs were hypothyroidism (6.9%), asthenia (4.4%), diarrhea (3.4%; assumed to be immune related based on response to empirical steroid treatment), rash (2.9%), hyperthyroidism (2.9%), dyspnea (2.9%), pruritus (2.5%), and pneumonitis (2.5%). Grade ≥ 3 irAEs were reported for 6.9% of patients (14/204). Pneumonitis was the only grade ≥ 3 treatment-emergent irAE that occurred in two patients (1.0%); all other grade ≥ 3 irAEs occurred in a single patient (0.5%). One patient receiving 240 mg Q2W experienced serious autoimmune colitis and discontinued study treatment.

IRRs were reported for 14.2% of patients (29/204). Median time to onset of first IRR was 15 (range, 1–148) days and median time to resolution of first IRR was 1 (range, 1–5) day. The majority of IRRs occurred after the first two doses. Two patients (1.0%) experienced grade ≥ 3 IRRs and one patient discontinued study treatment due to a grade 3 IRR of hypertension.

### Pharmacokinetics

**Fig. 2:**
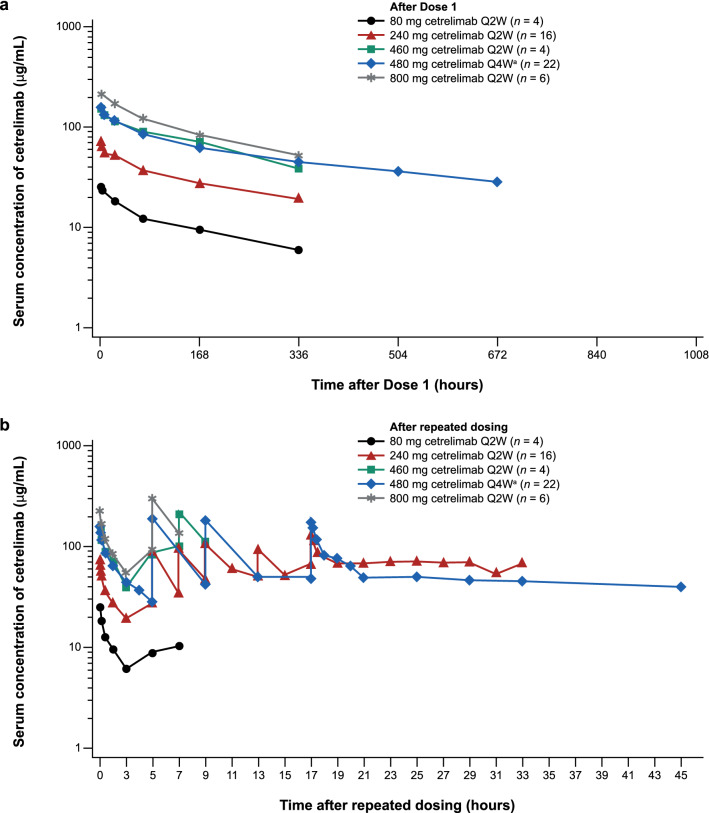
Mean (± SD) serum concentration–time curves **a** after the first cetrelimab dose, **b** after repeated cetrelimab doses. ^**a**^ Frozen and lyophilized drug product pooled

The pharmacokinetics of cetrelimab were linear and dose proportional across IV doses of 80, 240, 460, and 800 mg Q2W, with moderate variability after the first dose (Fig. 2a and Online Resource 1). The shape of the serum concentration–time profile was similar for all patients receiving cetrelimab 480 mg IV Q4W regardless of whether the drug product had been formulated as frozen liquid or lyophilized (Online Resource 2). Data from patients receiving 480 mg Q4W IV were pooled and analyzed (Online Resource 1).

Pharmacokinetic exposures [*C*_max_, trough concentration (*C*_trough_), AUC over a dosing interval (τ) (AUC_τ_)] increased after each repeated IV dose until steady state appeared to be reached after four doses with the Q4W schedule and seven doses with the Q2W schedule (Fig. 2b). After multiple IV doses of 240 mg Q2W and 480 mg Q4W cetrelimab, the steady-state accumulation ratios for these pharmacokinetic exposures were approximately 2.0- to 3.5-fold (Online Resource 3 and Online Resource 4).

The mean *C*_trough_ of 240 mg Q2W (66.9 µg/mL) was higher than the mean *C*_trough_ of 480 mg Q4W (48.2 µg/mL). The *t*_½_ calculated from NCA of steady-state pharmacokinetic data from the 480 mg Q4W groups (22.4 days) was consistent with the predicted *t*_½_ via popPK modeling (25.0 days). At Dose 9 of 240 mg Q2W during phase 2, the mean accumulation ratios of the *C*_trough_ and *C*_max_ were 2.86 and 1.84, respectively (*n* = 68). Both mean accumulation ratios were similar to those observed for 240 mg Q2W in phase 1 (3.29 and 2.05, respectively; *n* = 3).

Body weight and albumin were identified as two of the significant covariates for CL and body weight was identified as a significant covariate for V1 (data on file).

### Immunogenicity

The overall prevalence of anti-cetrelimab antibodies was 1.7% (3/182 patients with available data); 2.2% of patients in the phase 2 part of the study who were treated with IV cetrelimab 240 mg Q2W developed anti-cetrelimab antibodies (3/134 patients with available data). Anti-cetrelimab antibodies did not appear to affect cetrelimab pharmacokinetics (Online Resource 5).

### Pharmacodynamics

#### Biomarker expression

In the all-treated population, IP10 concentration was significantly increased (*P* = 0.012) over baseline on Day 43 (Online Resource 6). Significant elevation of IL2Ra chain was observed on Day 43 in patients with stable disease (*P* = 0.0006) and progressive disease (*P* < 0.0001), but not in patients with CR or PR following treatment with 240 mg Q2W cetrelimab.

#### Receptor occupancy

**Fig. 3:**
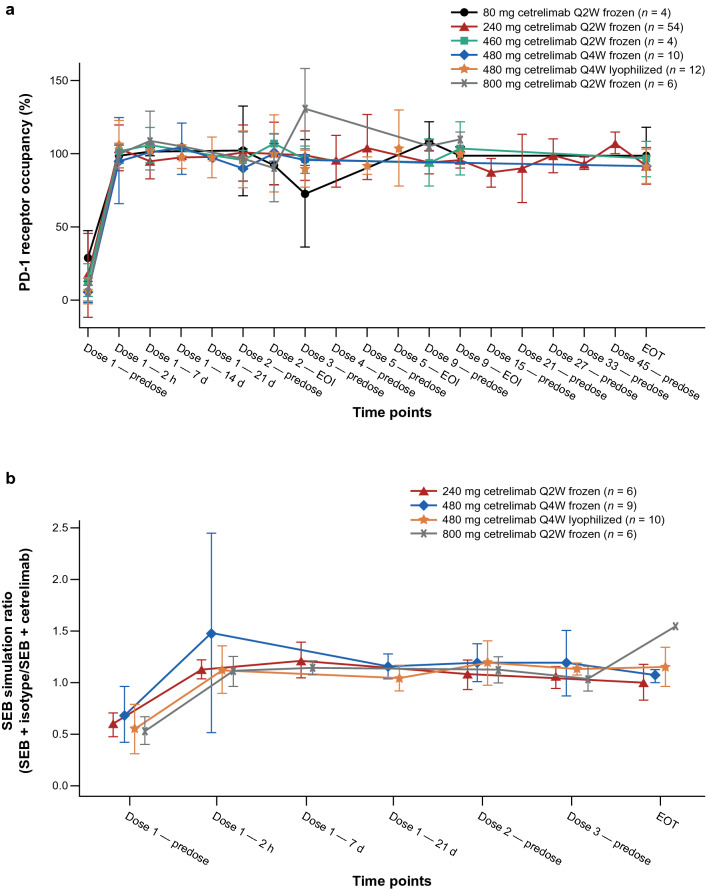
Pharmacodynamic effects of cetrelimab. **a** Mean (± SD) of PD-1 receptor occupancy over time. PD-1 receptor occupancy was measured by percent molecules of equivalent soluble fluorochrome (MESF) CD3^+^ in plasma by visit and dose level in the receptor occupancy analysis set. PD-1 receptor occupancy increased to 100% at all IV dose levels studied within 2 h post dose and remained at saturation at all time points during multiple doses). **b** Ratio of staphylococcal enterotoxin B (SEB)–stimulated interleukin-2 production in peripheral blood mononuclear cells treated with isotype versus cetrelimab. *EOI* end of infusion, *EOT* end of treatment, *IV* intravenous, *PD-1* programmed cell death protein-1, *Pre* predose, *Q2W* every 2 weeks, *Q4W* every 4 weeks

PD-1 RO saturation was achieved at the first time point when RO samples were collected, 2 h post-EOI. Similar levels of PD-1 RO saturation on CD3^+^ T cells were observed across all dose levels throughout treatment and at the EOT visit after cetrelimab discontinuation (Fig. 3a).

#### Ex vivo pharmacodynamics modulation

Cetrelimab treatment induced maximum IL-2 expression (stimulation ratio = 1) across all doses (240 mg Q2W, 480 mg Q4W, 800 mg Q2W) tested (Fig. 3b), indicating that maximal inhibitory activity of PD-1 was achieved and maintained throughout the dosing period across all doses, extending to 30 days after cessation of cetrelimab treatment.

### Efficacy

In the all-treated population from both phases of LUC1001, the ORR was 18.6% (38/204) and the CBR was 31.3% (64/204). Six patients (2.9%) had CR, 32 (15.7%) had PR, and 36 (17.7%) had stable disease. Median PFS was 2.8 (95% CI 1.9–3.7) months and median OS was 17.8 (95% CI 11.9–22.6) months (Online Resource 7 and Online Resource 8).

#### Non-small-cell lung cancer cohort

Of 35 patients with NSCLC in phase 1 and phase 2 included in the clinical analysis, 71.4% were diagnosed with adenocarcinoma, 22.9% with squamous cell carcinoma, 1 with large-cell carcinoma, and 1 with “other.” At screening, 94.3% of patients had stage IV NSCLC, and 97.1% had previously received chemotherapy. Mutation status was known for 15 patients (42.9%): a *KRAS* mutation was identified in 3 patients (8.6%), an *EGFR* mutation in 3 (8.6%), and other mutations in 9 (25.7%). Nineteen patients (54.3%) expressed high levels of PD-L1 and would have been eligible to receive PD-1 inhibitor therapy in clinical practice.

**Fig. 4:**
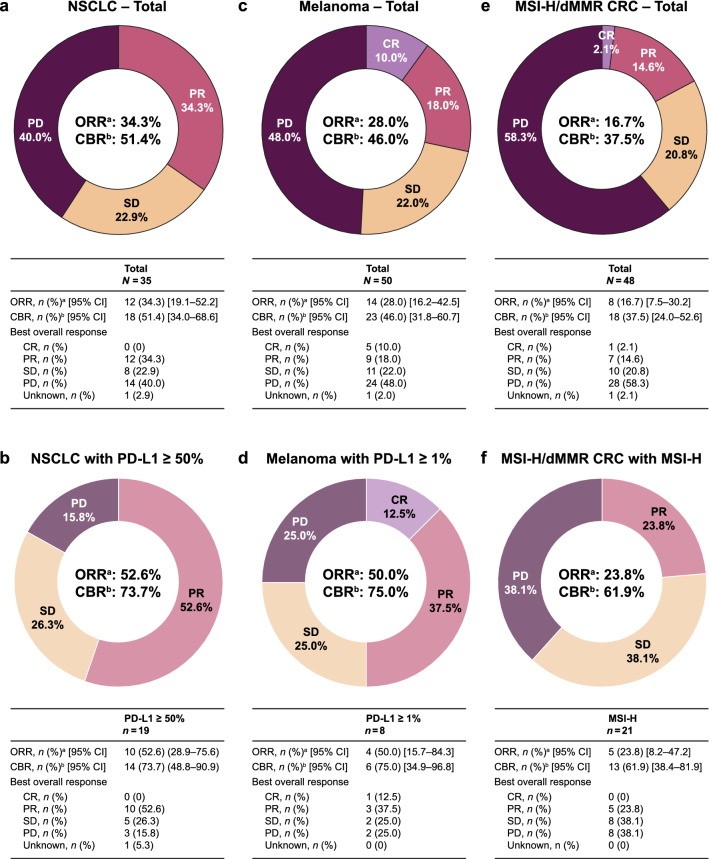
Efficacy measures in patients with **a**, **b** NSCLC (total and PD-L1 ≥ 50%), **c**, **d** melanoma (total and PD-L1 ≥ 1%), and **e**, **f** MSI-H/dMMR CRC (total and MSI-H). ^a^ORR is defined as the percentage of all treated patients with CR or PR. ^b^CBR is defined as the percentage of all treated patients with CR, PR, or SD (≥ 24 weeks after first study drug). *CBR* clinical benefit rate, *CI* confidence interval, *CR* complete response, *CRC* colorectal cancer, *dMMR* DNA mismatch repair deficient, *MEL* melanoma, *MSI*-*H* microsatellite instability–high, *NSCLC* non-small-cell lung cancer, *ORR* overall response rate, *PD* progressive disease, *PD-L1* programmed death ligand 1, *PR* partial response, *SD* stable disease

The median follow-up was 15.7 (range, 0.3–25.4) months. In all 35 patients, the ORR was 34.3% and the CBR was 51.4%. In the subgroup of 19 patients with PD-L1-high (≥ 50% PD-L1^+^) tumors, the ORR was 52.6% and the CBR was 73.7%. The overall median duration of treatment was 7.0 (range, 0.0–24.7) months for all treated patients and longer [10.7 (range, 1.0–24.7) months] among patients with PD-L1 high tumors. Median DOR could not be estimated, because there was an insufficient number of progression events at data cutoff. For all treated patients, median PFS was 7.4 [95% CI 2.43–not estimable (NE)] months and the median OS was 22.4 (95% CI 9.95–NE) months (Online Resource 7 and Online Resource 8). Response and duration of treatment for individual patients are shown in Online Resource 9 and Fig. 4.

#### Melanoma cohort

Fifty patients with melanoma enrolled in phase 1 and phase 2 combined, including six with uveal melanoma. Of these, 37 (74.0%) had previously received systemic treatment. Prior treatments included ipilimumab (38.0%), *BRAF/MEK*-targeted therapy (26.0%), IFN (24.0%), and chemotherapy (36.0%). All patients had stage IV melanoma at screening except one patient (2.0%) with stage III. *BRAF* mutations were found in 19 patients (45.2%) and PD-L1 status was positive (> 1%) for 8 (16.0%) patients. The median duration of follow-up for all patients was 16.6 (range, 0.7–25.1) months. The overall median duration of treatment was 5.5 (range, 0.0–25.0) months for all treated patients.

In all 50 patients, the ORR was 28.0% and the CBR was 46.0%. The six patients with uveal melanoma had progression as best response. Among the eight patients who were PD-L1^+^ (PD-L1 IHC ≥ 1%), the ORR was 50.0% and the CBR was 75.0%. Median DOR and OS could not be estimated (Online Resource 7). The median PFS for all treated patients was 5.4 (95% CI 2.73–9.95) months (Online Resource 8). Response and duration of treatment for individual patients are shown in Online Resource 10 and Fig. 4.

#### MSI-H/dMMR CRC cohort

Of 48 patients with CRC enrolled in phase 1 and 2, 21 were centrally determined to be MSI-H by IHC. Twelve patients were normal (microsatellite stable) by central laboratory and nine could not be evaluated by the central laboratory because of lack of control sample or insufficient tumor sample. Twenty-seven patients (56.3%) were dMMR by local laboratory. Overall, MSI-H testing was highly variable, with ~ 50.0% concordance between local and central laboratories. At screening, all patients with CRC had stage IV disease, and all had received prior systemic therapy; 46 patients (95.8%) received chemotherapy and one (2.1%) received *BRAF/MEK*-targeted therapy.

Patients with MSI-H CRC had a median treatment duration of 9.9 (range, 0.0–16.1) months, while the all-treated CRC population had a median treatment duration of 2.4 (range, 0.0–19.9) months. The median follow-up was 12.1 (range, 0.3–24.3) months for the all-treated population and 12.3 (range, 0.9–16.3) months for those with MSI-H CRC.

In all 48 patients with CRC, the ORR was 16.7% and the CBR was 37.5%. In patients with MSI-H CRC, the ORR was 23.8%, with PR being the best overall response for all five. The CBR was 61.9% in the MSI-H group. Median DOR and OS could not be estimated (Online Resource 8). Response and duration of treatment for individual patients with CRC are shown in Online Resource 11 and Fig. 4. The median PFS for all treated patients with CRC was 2.1 (95% CI 1.84–7.26) months (Online Resource 7).

## Discussion

In this first-in-human, phase 1/2 study, the safety, pharmacokinetics, pharmacodynamics, and biomarkers of cetrelimab were thoroughly characterized in PD-1/PD-L1 inhibitor–naïve patients with advanced or refractory solid tumors. Detailed characterization of cetrelimab monotherapy is essential for the selection of dosing regimens to accommodate potential schedules of combination partners. In phase 1 of LUC1001, cetrelimab was well tolerated across the IV doses of 80, 240, 460, or 800 mg Q2W or 480 mg Q4W. The safety profile of cetrelimab was consistent with that of other anti-PD-1 antibodies. As expected with immune checkpoint inhibitors, immune-related AEs were observed and they were managed with corticosteroids and treatment interruption. Only two DLTs occurred during the dose-escalation phase. Across phases 1 and 2, although 137 patients (67.2%) experienced TEAEs deemed possibly treatment related, most were managed by dose interruption, and only 12 patients (5.9%) experienced treatment-related AEs resulting in treatment discontinuation. No unexpected safety signals were observed. The prevalence of IRRs was higher for cetrelimab (14.2%), as reported by the investigators, compared with that reported for pembrolizumab (0.2%) and nivolumab (6.4%) [24–26]. Notably, the majority of cetrelimab IRRs were grade 1–2 and occurred after the first two doses. Other studies with cetrelimab using the lyophilized formation have not reported high frequencies of IRRs [27].

Serum cetrelimab steady-state *C*_trough_ for IV doses of cetrelimab 240 mg Q2W (66.9 µg/mL) and 480 mg Q4W (48.2 µg/mL) derived from the simulations, based on the popPK model parameters and associated interpatient variability, were similar and in range with other PD-1 inhibitors such as nivolumab [28–30] (56.5 µg/mL) and pembrolizumab [31–33] (23.3 µg/mL) at their respective approved clinical doses. The two cetrelimab regimens achieved the same total dose exposure, while demonstrating acceptable safety and sufficient pharmacokinetics/pharmacodynamics coverage to ensure RO saturation throughout the dosing interval. Furthermore, the clearance of cetrelimab at steady state (8. 6 mL/h in the 240 mg IV Q2W group, 9.8 mL/h in the 480 mg IV Q4W group) was similar and in range with that of nivolumab [30] (9.5 mL/h) and pembrolizumab [33] (9.2 mL/h) at their respective approved clinical doses. The median *t*_½_ of cetrelimab was aligned between NCA (22.4 days) and the popPK model (25.0 days) and conforms to the expected behavior of enhanced neonatal FC receptor–mediated recycling for IgG antibodies at around 3 weeks [34]. The reported terminal *t*_½_ was 25 days for nivolumab [28] and in the range of 14–22 days for pembrolizumab [35]. Based on the totality of safety, pharmacokinetics, pharmacodynamics, biomarkers, and efficacy data, the RP2D for cetrelimab may be 240 mg Q2W or 480 mg Q4W. The different dosing intervals provide flexibility in coordinating combination treatment; patients only received 240 mg Q2W in the phase 2 study to minimize sample size.

A relatively flat dose–response relationship has been demonstrated for two approved PD-1 inhibitors (nivolumab and pembrolizumab), suggesting a relatively broad therapeutic window (around 1–10 mg/kg) for the anti-PD-1 mechanism of action [28, 36, 37]. Nivolumab 480 mg Q4W was shown to be equivalent in clinical safety and efficacy to its previously approved 240 mg Q2W dosing schedule and has been approved as an alternate dosing schedule for most oncology indications in the United States [1, 24, 38]. For pembrolizumab, based on exposure–response modeling, the dosing interval has been extended to 400 mg Q6W [39] and longer dosing intervals may also be considered [25].

Anti-cetrelimab antibodies were detected in a small proportion of patients in phase 1 (1.7%) and phase 2 (2.2%) and did not appear to affect the pharmacokinetics of cetrelimab. Therefore, available data suggest that anti-cetrelimab antibodies do not impact clinical activity.

Preliminary efficacy data reported suggest that the efficacy profile of cetrelimab may be consistent with known profiles of PD-1 inhibitors in melanoma [40–45], PD-L1-high NSCLC [7, 11, 46–49], and MSI-H CRC [14, 15, 50]. Focusing on these tumor types allowed for a better point estimate on the response rate to be observed with cetrelimab in the sample sizes selected for this study. Compared with all treated patients, ORRs were high in subgroups with tumors carrying predictive biomarkers. This study supports existing evidence that suggests MSI testing is highly variable and should be standardized to improve the reliability of determining MSI-H among patients with CRC [51].

In summary, the phase 1/2 LUC1001 study characterized the safety, pharmacokinetics, pharmacodynamics, and efficacy data of cetrelimab in patients for the first time to support cetrelimab dose schedules of IV 240 mg Q2W and 480 mg Q4W. It is important to acknowledge that multiple PD-1 agents have already been approved for the treatment of PD-1/PD-L1 inhibitor–naïve patients with advanced or refractory solid tumors; cetrelimab is currently being developed to support combination studies that are ongoing. Furthermore, data from the phase 1/2 LUC1001 study cannot be directly compared with those from other trials at this time due to the small sample size and study design that is not controlled with an active comparator. The data reported here are critical for understanding cetrelimab in a clinical setting, and these findings are consistent with results with other approved PD-1 inhibitors. Based on the preliminary antitumor activity of cetrelimab in patients with advanced solid tumors in LUC1001 [52], studies are ongoing to evaluate the safety and efficacy of cetrelimab in combination with intravesical gemcitabine (TAR-200; JNJ-17000139-AAC) in non-muscle-invasive bladder cancer (NCT04640623) and in combination with erdafitinib in patients with urothelial carcinoma carrying *FGFR* alterations (NCT03473743), as chemotherapy or targeted therapy can release tumor neoantigens and prime the tumor microenvironment for immune response by a PD-1 inhibitor. These studies, however, are required to determine the safety and efficacy of such combinations based on the unpredictability in treating cancer and delivering meaningful new treatment options to patients.

## Supplementary Information

Below is the link to the electronic supplementary material.

Supplementary file1 (DOCX 654 KB)

## Declarations

### Availability of data and materials

Janssen Pharmaceutical Companies of Johnson & Johnson’s data sharing policy is available at https://www.janssen.com/clinical-trials/transparency. As noted on this site, requests for study data access can be submitted through Yale Open Data Access (YODA) Project site at http://yoda.yale.edu.
