# Supplementary material for: First-in-human, open-label, phase 1/2 study of the monoclonal antibody programmed cell death protein-1 (PD-1) inhibitor cetrelimab (JNJ-63723283) in patients with advanced cancers
Source: Cancer Chemother Pharmacol. 2022 Mar 17;89(4):499–514. doi: 10.1007/s00280-022-04414-6 (PMC8956549; doi:10.1007/s00280-022-04414-6)
Supplement: Supplementary file 1 — Supplementary file1 (DOCX 654 KB) [file 280_2022_4414_MOESM1_ESM.docx]

SUPPLEMENTARY MATERIAL

**First-in-human, open-label, phase 1/2 study of the monoclonal antibody PD-1 inhibitor cetrelimab (JNJ-63723283) in patients with advanced cancers**

Enriqueta Felip^1^ ∙ Victor Moreno^2^ ∙ Daniel Morgensztern^3^ ∙ Giuseppe Curigliano^4^ ∙ Piotr Rutkowski^5^ ∙ José Manuel Trigo^6^ ∙ Aitana Calvo^7^ ∙ Dariusz Kowalski^5^ ∙ Diego Cortinovis^8^ ∙ Ruth Plummer^9^ ∙ Michele Maio^10^ ∙ Paolo A. Ascierto^11^ ∙ Vladimir I. Vladimirov^12^ ∙ Andres Cervantes^13^ ∙ Enrique Zudaire^14^ ∙ Anasuya Hazra^14^ ∙ Huybrecht T’jollyn^15^ ∙ Nibedita Bandyopadhyay^16^ ∙ James G. Greger^14^ ∙ Edward Attiyeh^14^ ∙ Hong Xie^14^ ∙ Emiliano Calvo^17,^^[[1]](#footnote-1)^*

^1^ Thoracic Cancer Unit, Oncology Department, Vall d’Hebron University Hospital, Vall d’Hebron Institute of Oncology, Barcelona, Spain

^2^ Phase 1 Trials Unit, START MADRID-FJD, Hospital Fundación Jiménez Díaz Medical Oncology Division, Madrid, Spain

^3^ Division of Oncology, Section of Medical Oncology, Washington University School of Medicine, St. Louis, MO, USA

^4^ Division of Early Drug Development, European Institute of Oncology, IRCCS and University of Milano, Milan, Italy

^5^ Department of Soft Tissue/Bone Sarcoma and Melanoma, Maria Sklodowska-Curie National Research Institute of Oncology, Warsaw, Poland

^6^ Department of Medical Oncology, Hospital Universitario Virgen de la Victoria y Regional, Malaga, Spain

^7^ Oncology Service, Hospital General Universitario Gregorio Maranon, Madrid, Spain

^8^ Oncology Unit, San Gerardo Hospital, Monza, Italy

^9^ Sir Bobby Robson Unit, Northern Centre for Cancer Care, Newcastle, Newcastle Hospitals NHS Trust and Newcastle University, UK

^10^ Oncological Immunotherapy, Azienda Ospedaliera Universitaria Senese, Siena, Italy

^11^ Unit of Melanoma, Cancer Immunotherapy and Development Therapeutics, Istituto Nazionale Tumori- IRCCS-Fondazione Pascale, Napoli, Italy

^12^ Pyatigorsky Oncology Dispensary, Pyatigorsk, Russia

^13^ Medical Oncology Department, INCLIVA Biomedical Research Institute. University of Valencia, Valencia, Spain

^14^ Janssen R&D, Spring House, PA, USA

^15^ Janssen R&D, Beerse, Belgium

^16^ Janssen R&D, Raritan, NJ, USA

^17^ START Madrid-CIOCC, Centro Integral Oncológico Clara Campal Medical Oncology Division, Sanchinarro University Hospital, Madrid, Spain

CONTENTS

[**Online Resource 1** Pharmacokinetic results after the first IV administration of cetrelimab 80, 240, 460, 480, and 800 mg 1](#_Toc95810209)

[**Online Resource 2** Mean (SD) serum concentration–time profiles of cetrelimab at 480 mg IV Q4W administration of frozen liquid, lyophilized drug and as pooled formulation 2](#_Toc95810210)

[**Online Resource 3** Pharmacokinetic results at steady state (Dose 5 for 480 mg Q4W IV; Dose 9 for 240 mg Q2W IV) 3](#_Toc95810211)

[**Online Resource 4** Serum concentrations of cetrelimab predose and at end of infusion after each IV 240 mg dose (phase 2) 4](#_Toc95810212)

[**Online Resource 5** Summary of serum cetrelimab concentrations by anti-cetrelimab antibodies status in patients who received cetrelimab 240 mg IV Q2W in the phase 2 part of LUC1001 5](#_Toc95810213)

[**Online Resource 6** Pharmacodynamic effects of cetrelimab on (**a**) IP10 and (**b**) IL2Ra 6](#_Toc95810214)

[**Online Resource 7** Progression-free survival for patients with NSCLC, melanoma, and MSI-H / dMMR CRC 7](#_Toc95810215)

[**Online Resource 8** Overall survival for patients with NSCLC, melanoma, and MSI-H / dMMR CRC 8](#_Toc95810216)

[**Online Resource 9** Response and duration of treatment for patients with NSCLC (*N* = 35) 9](#_Toc95810217)

[**Online Resource 10** Response and duration of treatment for patients with melanoma (*N* = 50) 10](#_Toc95810218)

[**Online Resource 11** Response and duration of treatment for patients with MSI-H/dMMR colorectal cancer (*N* = 48) 11](#_Toc95810219)

**Online Resource 1** Pharmacokinetic results after the first IV administration of cetrelimab 80, 240, 460, 480, and 800 mg

|  | IV 80 mg *n* = 4^a^ | IV 240 mg *n* = 16^a,b^ | IV 460 mg *n* = 4^a^ | IV 480 mg *n* = 22^a,b,c^ | IV 800 mg *n* = 6^a,d^ |
| --- | --- | --- | --- | --- | --- |
| *C*_max_, µg/mL, mean (SD) | 24.7 (3.6) | 72.7 (17.8) | 148.0 (8.4) | 164.0 (40.9) | 227.0 (53.9) |
| *T*_max_, h, median (range) | 2.0 (1.0–3.1) | 1.2 (1.0–5.2) | 2.4 (1.3–3.4) | 3.0 (1.0–5.1) | 3.2 (1.0–6.1) |
| AUC_0–336h_, µg∙h/mL, mean (SD) | 3630.0 (147.0) | 10,918.0 (2850.0) | 24,907.0 (5089.0) | 23,976.0 (7157.0) | 33,397.0 (4209.0) |
| AUC_0–672h_, µg∙h/mL,  mean (SD) | — | — | — | 36,167.0 (11126.0) | — |
| AUC_last_, µg∙h/mL,  mean (SD) | 3630.0 (147.0) | 11,167.0 (2912.0) | 24,907.0 (5089.0) | 36,953.0 (11427.0) | 33,397.0 (4209.0) |
| *t*_last_, h, median  (range) | 340.0  (336.3–361.3) | 337.4  (311.4–723.0) | 338.1 (334.8–358.5) | 673.5 (600.1–1750.6) | 339.9  (336.1–363.4) |
| *C*_max,dn_, µg/mL/mg, mean (SD) | 0.31 (0.05) | 0.30 (0.07) | 0.32 (0.02) | 0.34 (0.09) | 0.28 (0.07) |
| AUC_0–336h,dn_, µg∙h/mL/mg, mean (SD) | 45.4 (1.8) | 45.5 (11.9) | 54.1 (11.1) | 49.9 (14.9) | 41.7 (5.3) |
| AUC_0–672h,dn_, µg∙h/mL/mg, mean (SD) | — | — | — | 75.3 (23.2) | — |
| *t*_½_, h, mean (SD) | 268.9 (61.9) | 245.5 (49.3) | 234.0 (100.0) | 414.7 (111.7) | 251.6 (106.1) |

*AUC* area under the serum concentration versus time curve, *AUC_0–336h_* AUC from time 0–336 hours, *AUC_0–672h_* AUC from time 0–672 hours, *AUC_last_* AUC from time 0 to time of last measurable concentration, *C_max_* maximum observed serum concentration, *dn* dose normalized, *IV* intravenous, *SC* subcutaneous, *SD* standard deviation, *t_½_* half-life, *t_last_* time of last measurable concentration, *T_max_* time to *C*_max_

^a^ An accurate determination of clearance was not possible in ≥ 3 patients; therefore, descriptive statistics for clearance after first dose are not reported

^b^*n* = 14 for AUC_0–336h_ and AUC_0–336h_, _dn_, *n* = 15 for AUC_last_ and *t*_last_, *n* = 11 for *t*_½_

^c^Frozen liquid and lyophilized cetrelimab data pooled

*^d^ n* = 20 for AUC_0–672h_ and AUC_0–672h_, _dn_, *n* = 21 for AUC_last_ and *t*_last_, *n* = 18 for *t*_½_

^e^*n* = 5 for AUC_0–336h_, AUC_0–336h_, _dn_, AUC_last_, *t*_½_, and *t*_last_

**Online Resource 2** Mean (SD) serum concentration–time profiles of cetrelimab at 480 mg IV Q4W administration of frozen liquid, lyophilized drug and as pooled formulation


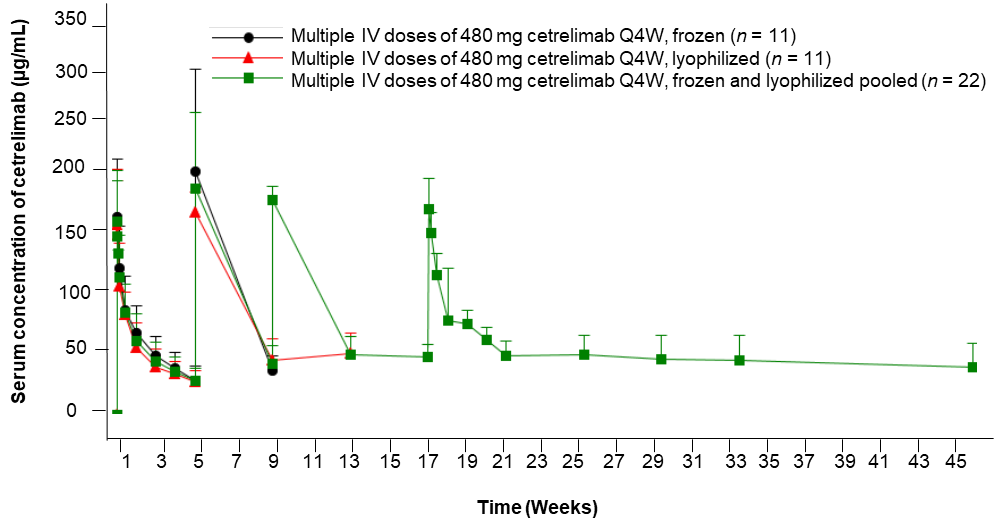


*IV* intravenous, *Q4W* every 4 weeks

**Online Resource 3** Pharmacokinetic results at steady state (Dose 5 for 480 mg Q4W IV; Dose 9 for 240 mg Q2W IV)

|  | 240 mg IV Q2W *n* = 3^a^ | 480 mg IV Q4W *n* = 4^b,c^ |
| --- | --- | --- |
| *C*_trough,_ µg/mL, mean (SD) | 66.9 (10.5) | 48.2 (11.3) |
| *C*_max_, µg/mL, mean (SD) | 139 (18.2) | 192 (33.7) |
| *T*_max_, h, median (range) | 0.8 (0.6–2.5) | 0.9 (0.6–1.5) |
| *AUC*τ, µg∙h/mL, mean (SD) | 28,768.0 (5694.0) | 50,808.0 (12296.0) |
| *AUC*_last_, µg∙h/mL, mean (SD) | 28,768.0 (5694.0) | 55,912.0 (14317.0) |
| *t*_last_, h, median (range) | 338.1 (335.0–338.3) | 686.4 (670.7–815.8) |
| *t*_½_, h, mean (SD) | — | 537.0 (69.2) |
| *CL*_ss_ mL/h, mean (SD) | 8.6 (1.6) | 9.8 (2.2) |
| *AR_C_*_trough_, _dose 5/dose 2_, mean (SD) | 3.3 (1.0) | 1.7 (0.3) |
| *AR_C_*_max_, _dose 5/dose 1_, mean (SD) | 2.1 (0.3) | 1.2 (0.2) |
| *AR*_AUC_𝜏, _dose 9/dose 2_, mean (SD) | 2.4 (0.4) | 1.5 (0.3) |
| *C*_trough_, _dn_, µg/mL/mg, mean (SD) | 0.28 (0.04) | 0.10 (0.02) |
| *C*_max, dn_, µg/mL/mg, mean (SD) | 0.58 (0.08) | 0.40 (0.07) |
| *AUC*τ, _dn_, µg∙h/mL/mg, mean (SD) | 120.0 (23.7) | 106.0 (25.6) |

^a^*n* = 4 for *C*_trough_, *C*_trough, dn_ and *AR_C_*_trough, dose 5/dose 2_

^b^Frozen liquid and lyophilized cetrelimab data pooled

*^c^* *n* = 3 for *C*L_ss_, *AUC*_𝜏_, *AUC*_𝜏, dn_ and *AR*_AUC𝜏, dose 9/dose 1_

*AR* accumulation ratio, *AUC* area under the serum concentration–time curve, *AUCτ* AUC at dosing interval (τ), *AUC_last_* AUC from time 0 to time of last measurable concentration, *CL_ss_* total systemic clearance at steady state, *C_max_* maximum observed serum concentration, *C_trough_* trough concentration, *dn* dose normalized, *IV* intravenous, *Q2W* every 2 weeks, *Q4W* every 4 weeks, *SD* standard deviation, *t*_½_ half-life, *t_last_* time of last measurable concentration, *T_max_* time to *C*_max_

**Online Resource 4** Serum concentrations of cetrelimab predose and at end of infusion after each IV 240 mg dose (phase 2)


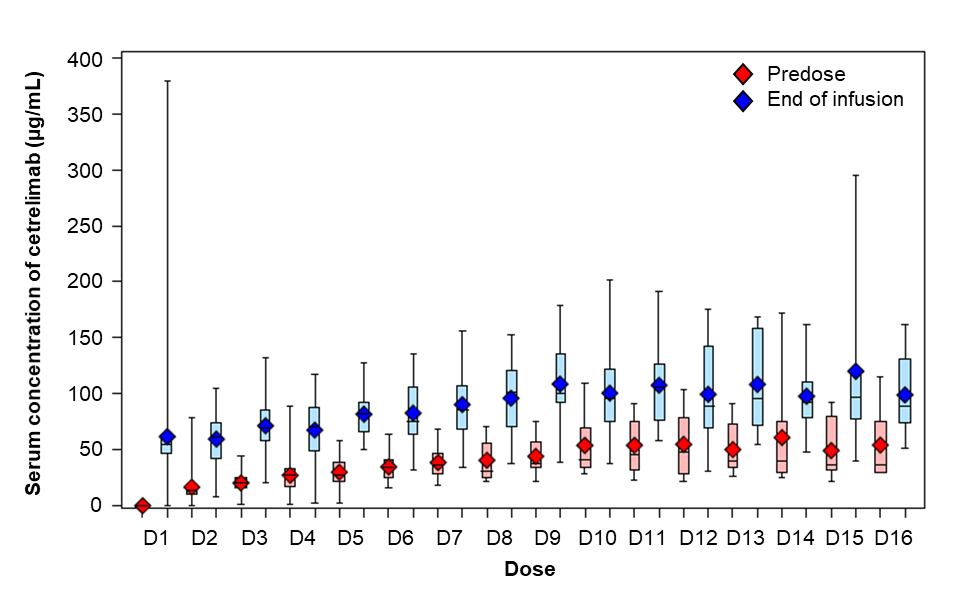


The bottom and top edges of the boxes indicate the interquartile range (the 25th and 75th percentiles). The diamonds indicate the mean value. The lines inside the boxes indicate the median value. The whiskers indicate the entire range of values. Boxes are shown only for time points where *n* ≥6

*D* dose, *IV* intravenous

**Online Resource 5** Summary of serum cetrelimab concentrations by anti-cetrelimab antibodies status in patients who received cetrelimab 240 mg IV Q2W in the phase 2 part of LUC1001

| Dose | Serum cetrelimab concentration, µg/mL, median (range) | |
| --- | --- | --- |
|  | Positive for anti-cetrelimab antibodies^a^ | Negative for anti-cetrelimab antibodies^b^ |
| Dose 1, Predose | BQL (BQL–BQL)  (*n* = 3) | BQL (BQL–BQL)  (*n* = 124) |
| Dose 1, 1 h | — (*n* = 2) | 58.8 (22.3–380.0)  (*n* = 75) |
| Dose 2, Predose | 18.5 (15.7–23.2) (*n* = 3) | 17.8 (4.6–79.0)  (*n* = 116) |
| Dose 2, 1 h | —  (*n* = 0) | 77.5 (8.6–919.0)  (*n* = 14) |
| Dose 3, Predose | —  (*n* = 2) | 25.4 (4.5–68.4)  (*n* = 106) |
| Dose 3, 1 h | —  (*n* =0) | 74.6 (19.4–406.0)  (*n* = 14) |
| Dose 4, Predose | 41.8 (29.1–49.8)  (*n* = 3) | 31.7 (BQL–90.7)  (*n* = 99) |
| Dose 4, 1 h | —  (*n* = 0) | 80.2 (14.9–146.0)  (*n* = 17) |
| Dose 5, Predose | 46.0 (33.9–59.4)  (*n* = 3) | 38.3 (5.0–150.0)  (*n* = 89) |
| Dose 6, 1 h | —  (*n* = 1) | 91.0 (52.8–169.0)  (*n* = 13) |

^a^ Patients positive for anti-cetrelimab antibodies showed an increase in anti-cetrelimab antibodies titers during the study

^b^ Patients who showed an increase in anti-cetrelimab antibodies titers during the study were excluded

*BQL* below quantification limit (<0.04000 μg/mL), *IV* intravenous, *Q2W* every 2 weeks

**Online Resource 6** Pharmacodynamic effects of cetrelimab on (**a**) IP10 and (**b**) IL2Ra


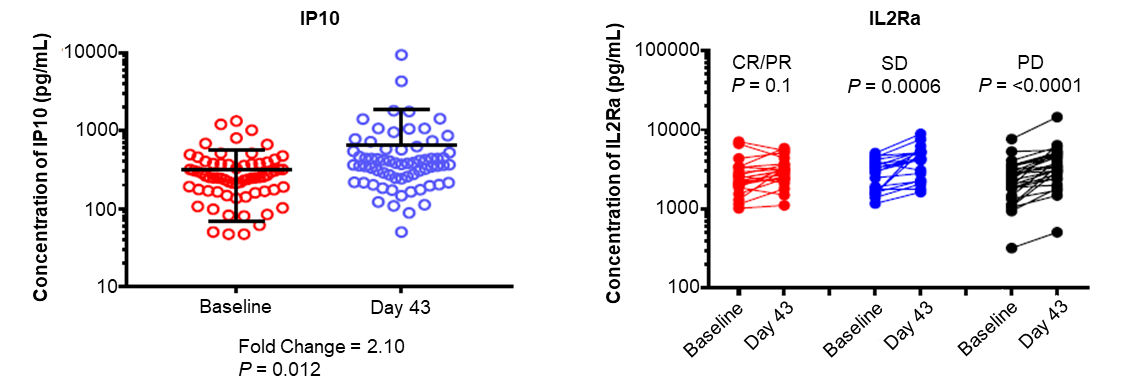


*CR* complete response, *IL2Ra* interleukin-2 receptor alpha, *IP10* interferon gamma-inducible protein 10, *PD* progressive disease, *PR* partial response, *SD* stable disease

**Online Resource 7** Progression-free survival for patients with NSCLC, melanoma, and MSI-H / dMMR CRC


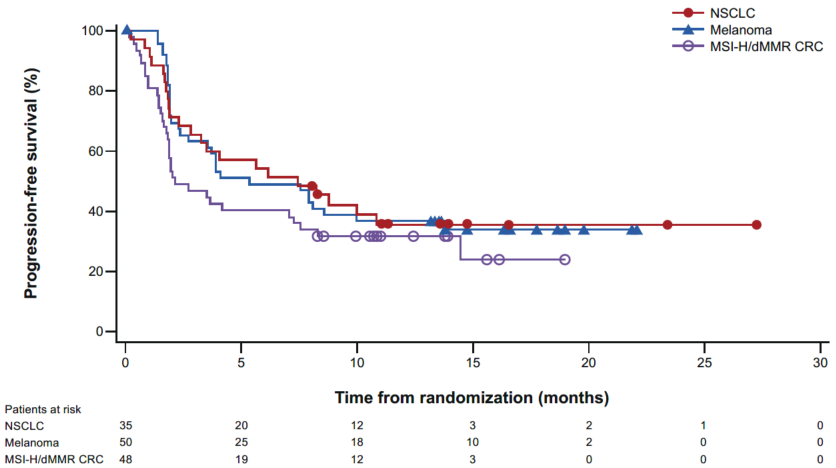


*CRC* colorectal cancer, *NSCLC* non-small-cell lung cancer, *dMMR* DNA mismatch repair deficient, *MSI-H* microsatellite instability–high

**Online Resource 8** Overall survival for patients with NSCLC, melanoma, and MSI-H / dMMR CRC

[Placeholder for figure]


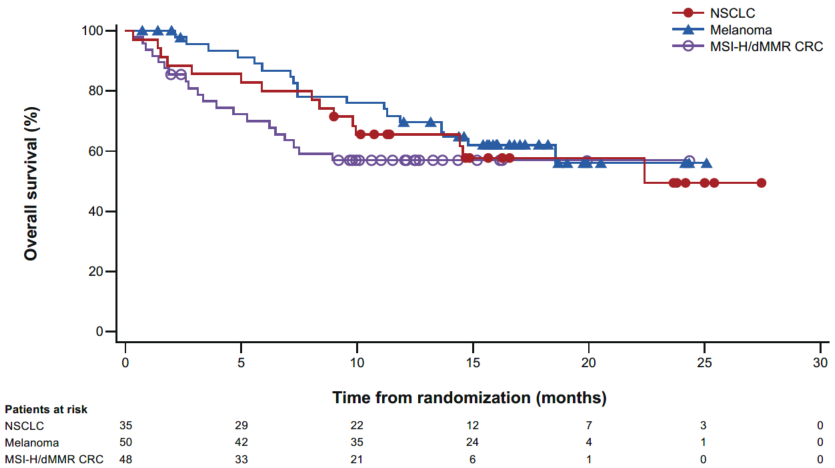


*CRC* colorectal cancer, *NSCLC* non-small-cell lung cancer, *dMMR* DNA mismatch repair deficient, *MSI-H* microsatellite instability–high

**Online Resource 9** Response and duration of treatment for patients with NSCLC (*N* = 35)


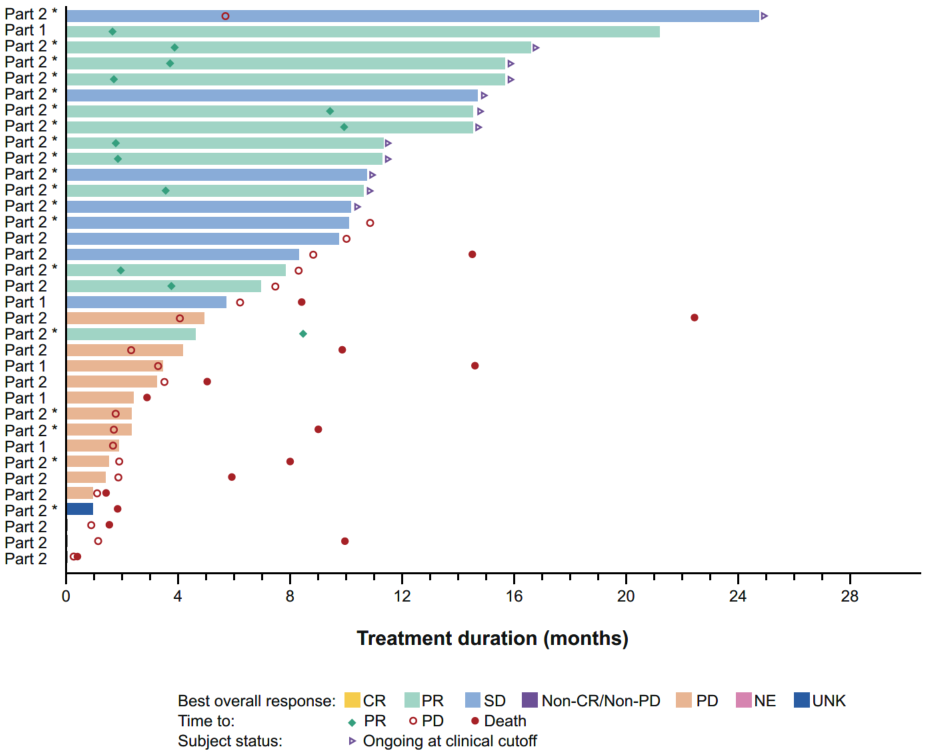


*Patient with PD-L1 high status

*CR* complete response, *NE* not evaluable, *NSCLC* non-small-cell lung cancer, *PD* progressive disease, *PD-L1* programmed death ligand 1, *PR* partial response, *SD* stable disease, *UNK* unknown

**Online Resource 10** Response and duration of treatment for patients with melanoma (*N* = 50)


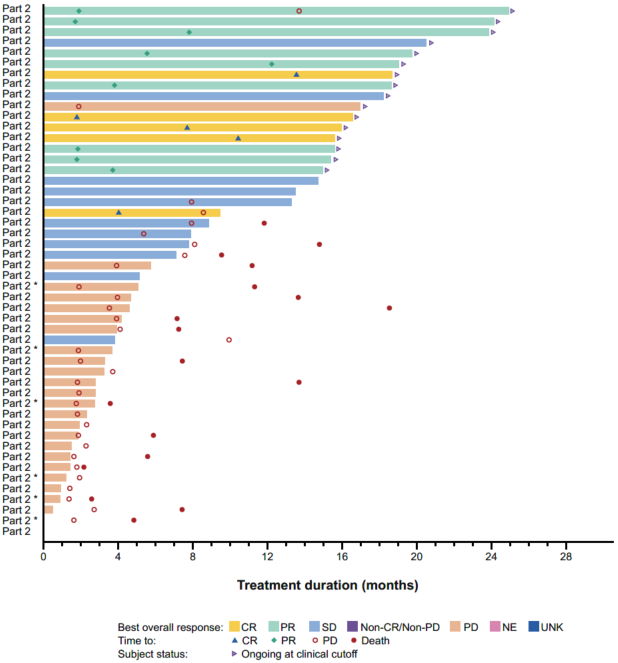


*Patient with PD-L1 high status

*CR* complete response, *MSI-H* microsatellite-high, *NE* not evaluable, *PD* progressive disease, *PD-L1* programmed death ligand 1, *PR* partial response, *SD* stable disease, *UNK* unknown

**Online Resource 11** Response and duration of treatment for patients with MSI-H/dMMR colorectal cancer (*N* = 48)


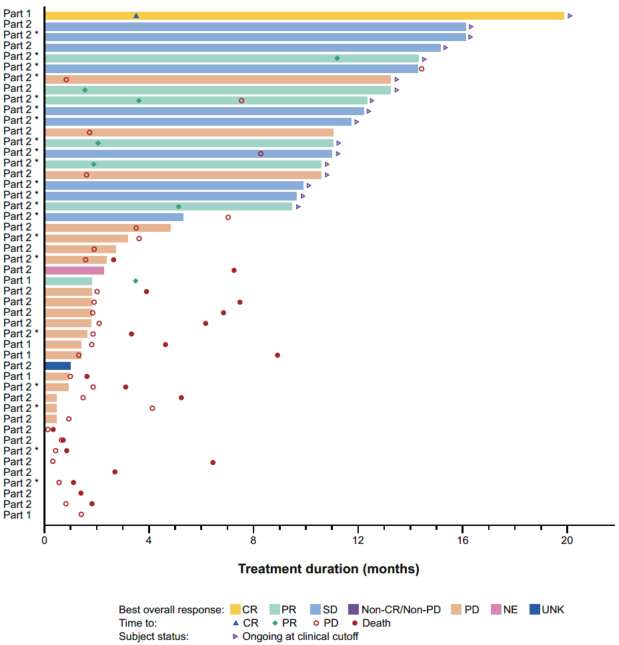


*Patient with centrally confirmed MSI-H status

*CR* complete response, *CRC* colorectal cancer, *dMMR* DNA mismatch repair deficient, *MSI-H* microsatellite instability–, *NE* not evaluable, *NSCLC* non-small-cell lung cancer, *PD* progressive disease, *PR* partial response, *SD* stable disease, *UNK* unknown

1. * Corresponding author at [emiliano.calvo@startmadrid.com](mailto:emiliano.calvo@startmadrid.com) [↑](#footnote-ref-1)
